# Diel variability of methane emissions from lakes

**DOI:** 10.1073/pnas.2006024117

**Published:** 2020-08-17

**Authors:** Anna K. Sieczko, Nguyen Thanh Duc, Jonathan Schenk, Gustav Pajala, David Rudberg, Henrique O. Sawakuchi, David Bastviken

**Affiliations:** ^a^Department of Thematic Studies–Environmental Change, Linköping University, 58183 Linköping, Sweden;; ^b^Department of Ecology and Environmental Sciences, Umeå Universitet, 901 87 Umeå, Sweden

**Keywords:** methane fluxes, diel variability, automated flux chambers, lake greenhouse gas emissions

## Abstract

Lakes are considered the second largest natural source of atmospheric methane (CH_4_). However, current estimates are still uncertain and do not account for diel variability of CH_4_ emissions. In this study, we performed high-resolution measurements of CH_4_ flux from several lakes, using an automated and sensor-based flux measurement approach (in total 4,580 measurements), and demonstrated a clear and consistent diel lake CH_4_ flux pattern during stratification and mixing periods. The maximum of CH_4_ flux were always noted between 10:00 and 16:00, whereas lower CH_4_ fluxes typically occurred during the nighttime (00:00–04:00). Regardless of the lake, CH_4_ emissions were on an average 2.4 higher during the day compared to the nighttime. Fluxes were higher during daytime on nearly 80% of the days. Accordingly, estimates and extrapolations based on daytime measurements only most likely result in overestimated fluxes, and consideration of diel variability is critical to properly assess the total lake CH_4_ flux, representing a key component of the global CH_4_ budget. Hence, based on a combination of our data and additional literature information considering diel variability across latitudes, we discuss ways to derive a diel variability correction factor for previous measurements made during daytime only.

## Significance

Methane (CH_4_) emissions from lakes are significant, yet still highly uncertain and a key bottleneck for understanding the global methane budget. Current lake flux estimates do not account for diel variability of CH_4_ flux. Here, we apply a high-resolution spatiotemporal measurement approach in multiple lakes and report extensive data on variability between day and night lake CH_4_ emissions. Our results demonstrate a clear and consistent diel pattern with more than twofold higher daytime fluxes. We show that it is critical to include diel variability to correctly estimate and extrapolate lake CH_4_ flux, and that present northern lake emissions may have been overestimated by 15%.

Methane (CH_4_) is one of the most important carbon-based greenhouse gases (GHG) (1). The relative increase in CH_4_ has been the highest of all GHGs over the industrial period (2). However, the rate of increase has been irregular for yet-unknown reasons, and the source/sink attribution of CH_4_ fluxes is unclear (3). Regarding natural sources of CH_4_, open water lake emissions have been identified as the second largest CH_4_ source (4). However, reported lake CH_4_ fluxes range from 40 to 200 Tg CH_4_ y^−1^, indicating a very large uncertainty (2, 5, 6). The lake CH_4_ fluxes also challenge the global CH_4_ budget of 500–700 Tg CH_4_ by generating a budget mismatch with larger total bottom-up flux estimates than fit in top-down budget constraints (3, 7). It is clear that more accurate estimates of lake CH_4_ fluxes are needed.

CH_4_ can be emitted from lakes to the atmosphere via several different pathways, including diffusion, ebullition, release from storage, and flux mediated by emergent plants (6). Main open water CH_4_ emission pathways—diffusion and ebullition—exhibit high spatiotemporal variability. Diffusive flux depends partly on the CH_4_ concentration gradient between the water and the air, and the turbulence in the surface water influencing gas transfer rates. Ebullition is highly dependent on CH_4_ production in the sediments and physical factors triggering the bubble release (e.g., variation in atmospheric and hydrostatic pressure). Accordingly, both diffusive flux and ebullition can be under strong influence of physical forcing, via e.g., pressure, wind, temperature, precipitation or radiation, which can operate on very short time scales (minutes to hours) and, thereby, potentially lead to a great CH_4_ flux variability within one diel cycle. Yet, lake CH_4_ fluxes have often been measured during daytime only. Short daytime measurements provide a snapshot of the full 24-h periods, resulting in limited understanding of the diel variability. The existing studies on diel lake flux variability are scarce, and although some of them demonstrate significant differences within a 24-h time span, the situation is unclear and the findings are often contradictory in terms of when fluxes are the greatest (8, 9) (see also *Results and Discussion*). In essence, most of the accumulated data are based on low-resolution measurements and limited study periods (10), rarely considering day-night variability. If significant differences between day and night CH_4_ emissions exist and are common, this could challenge and bias the existing large-scale estimates. Hence, understanding diel variability patterns of methane flux is essential for reliable upscaling and modeling of methane emissions from lakes.

In this study, we performed lake CH_4_ flux measurements resolving diel variability in four lakes with different physicochemical characteristics, which are situated along a climate gradient from the northern temperate to northern boreal zone (*SI Appendix*, Table S1 and Fig. S1). We used a combination of manual and automated flux chambers, which generated 4,580 individual flux estimates, covering in total 524 full chamber measurement days in the lakes. Our findings were compared with available literature to evaluate possible key drivers and implications of the observed diel patterns. This information was used to discuss ways to better consider diel variability when using former data and designing future measurements.

## Results and Discussion

### CH_4_ Flux Diel Pattern.

**Fig. 1.**
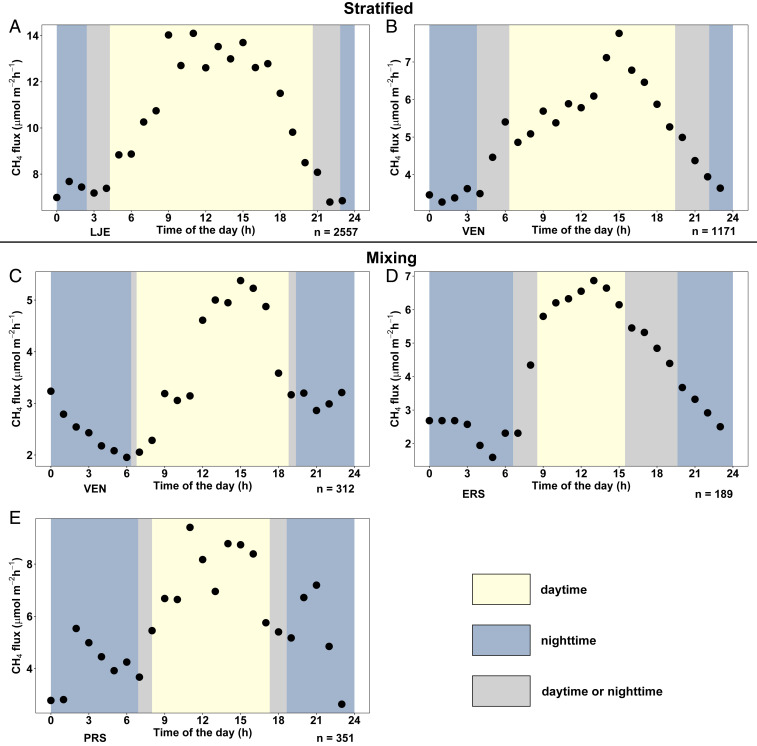
Mean (geometric weighted average; for details see *Materials and Methods*) of hourly CH_4_ fluxes over the full diel cycle (black dots) in the lakes LJE (*A*) and VEN (*B*) during stratified period, and in VEN (*C*), ERS (*D*), and PRS (*E*) during the mixing period (*n* = 4,580). Colored areas indicate day (yellow) or night (blue) during the time of the measurements, depending on the time of the year at a given location. Gray indicates times that during the sampling period was classified either as day or night.

The use of automated flux chambers (11) together with additional manual measurements resulted in a comprehensive dataset of CH_4_ emissions with a mean temporal resolution of 2 h (for details see *SI Appendix*, Table S2). Despite high flux fluctuations, there was a clear and consistent diel pattern with higher CH_4_ flux between 10:00 and 16:00 for most of the measurement days (Fig. 1). Statistical comparisons between time periods confirmed that CH_4_ emissions were significantly higher during daytime hours (10:00–16:00) compared to the nighttime (00:00–04:00) in the studied lakes (Fig. 1 *A*–*D*; *P* < 0.001, *P* < 0.001, *P* < 0.05, *P* < 0.01, respectively).

**Fig. 2.**
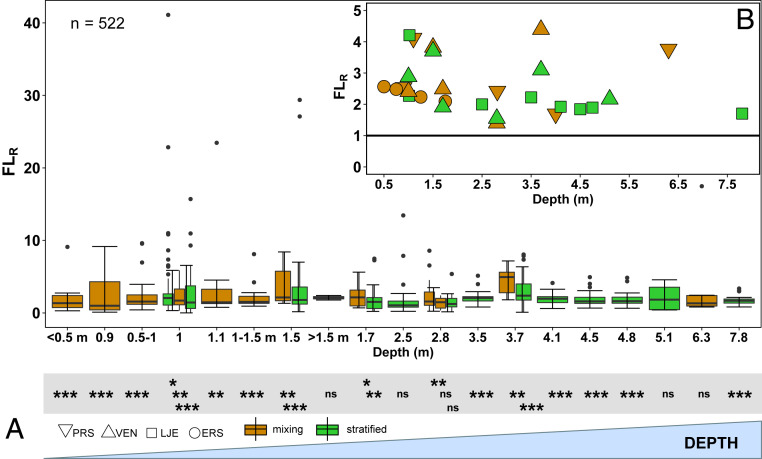
(*A*) Boxplots illustrating CH_4_ flux day:night ratio (FL_R_) at different depths in: PRS, ERS, LJE, and VEN lakes during mixing and the stratified period (brown and green boxes, respectively) (*n* = 522). The boundaries of each box plot indicate the 25th and 75th percentiles, points indicate outliers, and the solid line in each box marks the median. Asterisks (*) on the gray area below each boxplot illustrate if a given FL_R_ was statistically higher than 1 (Wilcoxon test), indicating significantly higher fluxes during the day; *P < 0.05, **P < 0.01, ***P < 0.001, not significant (ns). The *x* axis indicates the depths where CH_4_ fluxes were measured; (*B*) Average CH_4_ flux day:night ratio (FL_R_) for each lake and each depth during stratified and mixing period with each lake having different symbol shapes (*n* = 28). The figure shows that there was no clear depth dependency of FL_R_.

The measured CH_4_ day:night flux ratios (FL_R_) (Fig. 2*A*) were characterized by high variability, which was observed regardless of the lake or season (mixing or stratified). This is not surprising, as our methods captured both diffusion and ebullition with the latter resulting in high and episodic fluxes. Despite such variability and regardless of the depth, the highest FL_R_ were significantly higher than 1 (Fig. 2*A*), i.e., daytime fluxes were higher in most of the locations in the studied lakes. Moreover, the mean FL_R_ for each depth was always greater than 1 (Fig. 2*B*; 1.5–4.2 larger during the day vs. the night). This pattern did not differ significantly between mixing and stratified periods (*P* = 0.67).

**Fig. 3.**
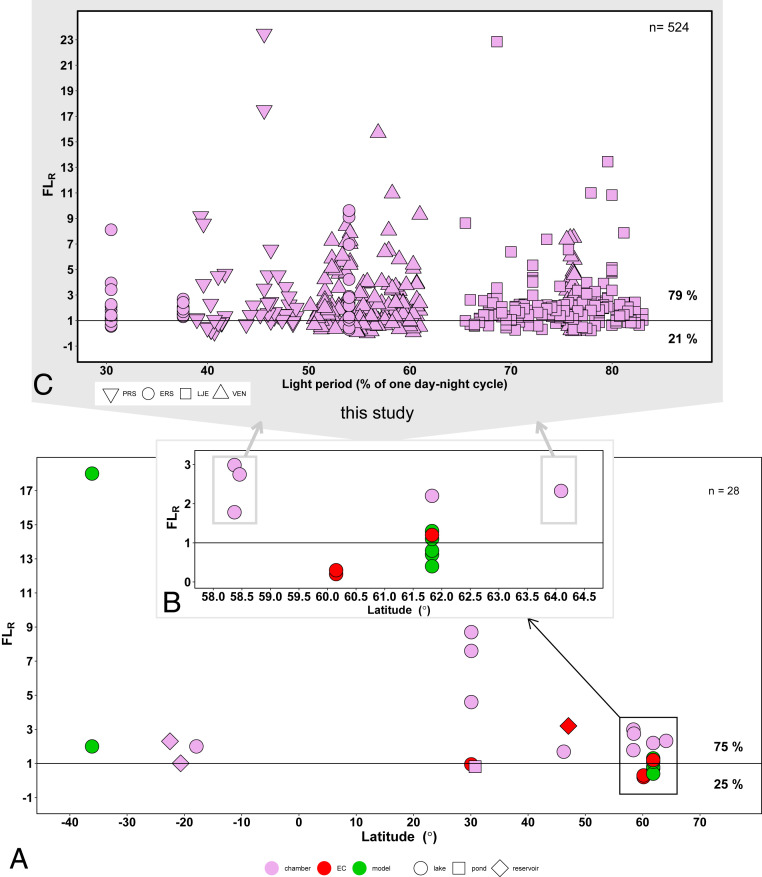
*A* and *B* show the average CH_4_ flux day:night ratio (FL_R_) in systems located in different latitudes (*n* = 28) (see also *SI Appendix*, Table S2) measured with different methods and including the average FL_R_ values for each lake from this study. Symbols indicate the system where CH_4_ fluxes were measured and colors represent methods used to obtain CH_4_ flux, including floating chambers (chamber), EC, and gas transfer models (model); for details see *SI Appendix*, Table S2. *C* shows individual FL_R_ (*n* = 524) in the lakes investigated in this study plotted against the length of the light period in each day-night cycle. Three values of FL_R_ (27.1, 29.4, 41.1) are not displayed to increase resolution of the figure. For details on day-night cycle definition, see *Materials and Methods*.

At a much larger latitudinal scale, considering the previous studies (*n* = 14) located between 36 °S and 64 °N (*SI Appendix*, Table S2), 75% of FL_R_ reported for lakes, ponds, and reservoirs (*n* = 25) were above value of 1 (i.e., greater emissions during the day; Fig. 3 *A* and *B*). This is similar to our study where 79% of the CH_4_ fluxes were also higher during the day (Fig. 3*C*). The FL_R_ was not related to the length of the light period during one day-night cycle, indicating that the diel variability of CH_4_ emissions is a general phenomenon, which may occur regardless of the latitude (thus the length of the daytime). Occasionally, however, we observed higher nighttime fluxes for some chambers, which suggests that with fewer and limited measurements our findings may have been less clear and relying only on fragmentary data may lead to biased conclusions.

The previous studies on full, 24-h methane flux cycles are limited and although some of them also demonstrated diel CH_4_ patterns, the findings were not consistent (12–19). Some of these studies rely on flux modeling from surface water concentrations and wind speed. In these cases, CH_4_ diel pattern typically reflects the diel variability in wind speed, which is often measured at much higher frequency than the water concentrations. Thus, lack of direct flux measurements in these cases creates difficulties to disentangle diel variability of CH_4_ flux from the wind speed patterns, and for this reason these studies may not be comparable with our data. The eddy covariance (EC) method is increasingly being used to assess CH_4_ fluxes from lakes, but the diel variability assessments based on EC are inconclusive; approximately half of the reported studies reported higher daytime fluxes, whereas the other half showed higher nighttime emissions (*SI Appendix*, Table S2). This can be due to the fact that the footprint of the measurements varies with wind speed and, hence, often vary between day and night. In addition, lake heat balances can interact with wind and generate lateral fluxes (local versions of land/sea breeze) in different directions during day and night. This creates difficulties to assess the extent to which day-night differences occur due to differences in footprint size and location or lateral fluxes. Moreover, EC estimates require wind-generated air turbulence. Nighttime is often characterized either by low wind speeds or by a stable atmospheric boundary layer. Under such conditions EC flux accuracy is reduced or flux estimation may not be possible, which could bias nighttime fluxes and, thereby, also the mean 24-h flux. The flux chamber studies provide the most consistent results, where seven of nine aquatic systems, which applied flux chambers, indicated higher daytime fluxes (this study excluded; *SI Appendix*, Table S2). Only two studies, which covered three 24-h periods showed higher emissions during nighttime or no clear diel pattern. Hence, most of the directly comparable data are in accordance with our results.

### Potential Drivers Influencing CH_4_ Diel Flux Patterns.

Temperature has been recognized as an important driver for CH_4_ production and flux (20). However, temperature changes between day and night noted in our study were very small, where day:night water temperature ratio in all of the lakes ranged from 0.997 to 1.006. Therefore, over single days, water temperature changes were unlikely to drive day-night variability in CH_4_. Convective mixing has been considered as a candidate for generating diel patterns in lake gas emissions (8, 21, 22). Nighttime surface water cooling may enhance formation of eddies of cooled higher density surface water, facilitating convective mixing, which, in turn, could increase CH_4_ emissions during the night. Convection has been suggested as important for facilitating high nighttime fluxes (23, 24) in wetlands and also implied as a potential mechanism for nighttime fluxes in a lake (8). However, the convective periods, generated by daytime heating of the water column followed by nighttime cooling do not appear to govern diel emission patterns in our study. Indeed, the temperature difference (ΔT) between water and air followed a diurnal cycle, but lower fluxes were observed during the night when ΔT was the highest, which would be expected to promote convective mixing (*SI Appendix*, Fig. S2). Although convective mixing could be influential during diel cycles with continuous very low wind speed (21, 25), its overall impact on CH_4_ diel flux patterns seems to be negligible compared to other mechanisms.

As seen in other lake ecosystems, short-term variations in CH_4_ flux can be driven by wind speed (26). This is in line with our data, which clearly showed that diel patterns of wind speed and CH_4_ flux coincided with each other (*SI Appendix*, Fig. S3). Elevated wind speed can augment diffusive flux by increasing the gas transfer coefficient and causing upwelling of gas-rich deeper water (27). Further, wind also results in enhanced wave turbulence, which, in turn, may cause pressure oscillation and currents at sediment surfaces and facilitate ebullition and sediment release of dissolved CH_4_. High wind events can be associated with passing low pressure weather systems, which have been shown to trigger ebullition (28). Thereby, the wind speed can influence CH_4_ fluxes in many ways. In this study, 70% of the FL_R_ > 1 were associated with greater wind speeds during the day (*SI Appendix*, Fig. S4). Hence, diel wind patterns were synchronous with diel flux patterns during most days. Although we observed a similar diel pattern of flux and wind, there was no clear linear correlation between wind speed and total flux, which was expected due to the irregularity in fluxes caused by the CH_4_ ebullition. In addition, 15% of the days with elevated wind speed during daytime did not correspond with highest daytime fluxes (*SI Appendix*, Fig. S4), which indicates that wind speed is not the only driver of importance for the diel flux pattern. Accordingly, our data indicate that wind speed is important but should not be considered as the sole driver of the diel flux differences. Considering that changes in atmospheric pressure have been previously highlighted as a trigger for elevated methane flux (28), pressure changes could potentially also generate diel flux patterns that are not consistent with wind-driven day-night patterns. To verify this hypothesis, we aimed to separate impact of wind from pressure effect on FL_R_ by investigating 24-h periods when pressure drop occurred, both during day and night, but daytime and nighttime wind speeds were similar (ratios ranged 0.7–1.3) (*n* = 68). Although daytime pressure drops were occasionally higher, there was no clear relation between day:night pressure drop ratio and FL_R._ Hence, we found no support for drops in atmospheric pressure driving day-night variability patterns.

Another environmental driver that could potentially impact diel CH_4_ flux dynamics is the intensity of the incoming radiation. It has been suggested that elevated photosynthetic active radiation (PAR) in the epilimnion may inhibit methane oxidation (29), implying that light could indirectly increase CH_4_ flux. Thus, a diel pattern of lower methane oxidation during the day due to the light inhibition could result in increased CH_4_ emissions in the daytime (30). In our study, hourly CH_4_ fluxes in all of the lakes were mirrored by PAR intensity (*SI Appendix*, Fig. S5). Moreover, for most lakes, intensity of PAR between 09:00 and 16:00 was well above 300 µmol·m^−2^·s^−1^, which is considered as a threshold for complete inhibition of growth and activity of methanotrophs (31). Thus, we cannot exclude that light inhibition of surface water methane oxidation influenced the observed diel flux patterns. However, it is unclear how far down into the water the light inhibition of methane oxidation would reach, and our data does not show any clear correlation between FL_R_ and light intensity (*SI Appendix*, Fig. S6), which could indicate that such light effects are limited or nonexisting.

Recent studies also proposed light-stimulated oxic surface water CH_4_ production associated with photosynthesis and chlorophyll a (chl*a*) (32, 33). Such oxic methane production has been suggested to have substantial input to the lake CH_4_ flux, especially during the times of the high phytoplankton growth (34), and might therefore also contribute to higher daytime fluxes. If this oxic CH_4_ surface water production would be important, a higher FL_R_ would be expected in lakes with higher chl*a* concentrations and at higher light availability. However, this was not apparent in our data. For instance, similar mean FL_R_ was observed in Venasjön (VEN) and Parsen (PRS) (2.8 and 2.9; respectively), while their mean chl*a* values were very different (*SI Appendix*, Table S1). Also, FL_R_ did not increase with light intensity (*SI Appendix*, Fig. S6). Accordingly, our data indicate that oxic surface CH_4_ production was not important for diel CH_4_ flux variability in the studied lakes, and altogether additional work would be needed to address where and when CH_4_ emissions could be affected by light.

### Importance of Diel Variability in Upscaling of CH_4_ Emissions from Lakes.

Currently, estimates of methane emissions from lakes often rely on measurements of CH_4_ fluxes, which were made during “office hours” (08:00–16:00) (35–37). Therefore, measuring a flux once or even several times, but limiting sampling to daytime only, will according to our results, in most cases (79% of the days) lead to overestimation of the total 24-h flux that particular day (Fig. 3). Consequently, using such results for large-scale estimations without consideration of diel variability may create a large bias.

As an attempt to estimate the influence of the daytime-only measurement on past estimates of northern lake CH_4_ emissions, we derived a correction factor. Our results and literature data from other direct flux measurements (flux chamber studies + EC) on average indicate 2.5 times higher daytime CH_4_ flux than the nighttime emission (Fig. 3 and *SI Appendix*, Table S2). Assuming that reported fluxes measured during daytime “office hours” are on an average 2.5 times higher than nighttime fluxes, a conversion factor (hereafter called “diel factor”) of 0.7 was derived in order to convert daytime-only measurements to full 24-h fluxes. Thus, average flux over 1 d (*F*_24h,mean_) accounting for 2.5 times higher flux during the day (*F*_day_) was calculated from the flux measured during the day (*F*_day_) as follows:

$$F_{\mathrm{24h,mean}}=\frac{F_{\mathrm{day}}+F_{\mathrm{night}}}{2}=\frac{F_{\mathrm{day}}+\left(F_{\mathrm{day}}/2.5\right)}{2}=0.7F_{\mathrm{day}}.$$

**Fig. 4.**
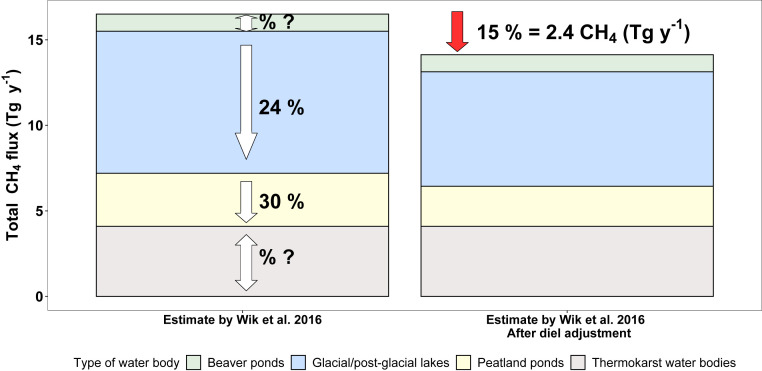
Large-scale estimates of CH_4_ emissions from inland waters north of ∼50° N (*Left*) (35). Percentages indicate the potential bias due to lack of diel variability considerations in the estimates used, and arrows show the direction of the bias (arrow downward indicate overestimate, i.e., that consideration of diel variability will reduce the flux estimate). *Right* shows the estimated total emissions after applying the correction for the diel variability to the data used in the Wik et al., 2016 study (35). The adjustment was applied to the glacial/postglacial lakes and to the peatland ponds; the estimate of CH_4_ flux from beaver ponds and thermokarst water bodies was not adjusted (see main text for details). Overall, the adjustment reduced the flux with 15% corresponding to 2.4 Tg CH_4_ y^−1^, indicated by the red arrow on top of *Right*.

In the Wik et al. (35) database for lake CH_4_ emissions from 733 lakes north of 50° N, we identified the studies relying on daytime-only measurements and applied the diel factor to those measurements. This resulted in 24% and 30% lower CH_4_ flux from glacial or postglacial lakes and peatland ponds, respectively (Fig. 4). The correction factor was not applied to beaver ponds and thermokarst lakes as flux chamber methods were not applied on these lake types and information on their diel flux variability was not available. The total northern lake CH_4_ flux estimate by Wik et al. (35), adjusted for diel variability in this way, was reduced by 15% compared to the previous assessment, corresponding to a decrease of 2.4 Tg CH_4_ per year (Fig. 4). Hence, it is clear that neglecting the diel variability in CH_4_ flux may lead to considerable bias in large-scale lake CH_4_ flux assessments. To our knowledge, none of the currently commonly cited global lake CH_4_ flux estimates (e.g., refs. 2–5) explicitly considers the influence of diel variability, yet they are based on a substantial proportion of daytime-only measurements. Hence, future work to include this aspect and to measure lake CH_4_ fluxes over full 24-h periods is needed.

Previous concerns regarding the lake CH_4_ flux uncertainty has emphasized that global fluxes may be underestimated due to the difficulty in properly measuring ebullition, and lack of consideration to hot spot areas (shallow and/or highly productive lakes) or hot flux moments (e.g., flux associated with lake circulation or ice-out events) (5, 6, 38, 39). Here, we highlight that diel variability is another factor influencing lake CH_4_ fluxes and that current flux estimates from the diel variability perspective alone may be overestimated. Future work to generate lake CH_4_ flux data, properly considering all of the identified causes for the present high uncertainty, is clearly needed. Failing to do this may result in a continued unknown systematic bias of regional and global estimates due to the lack of representative measurements. Therefore, accurate quantification of CH_4_ fluxes is a necessary step toward improved estimates and predictions of global CH_4_ emissions and to generate the knowledge needed to efficiently mitigate climate change (2).

## Materials and Methods

### Study Area.

Four lakes were selected, representing types often found in boreal and north temperate regions (*SI Appendix*, Fig. S1). All lakes are dimictic, and their coordinates and main characteristics are presented in *SI Appendix*, Table S1. Briefly, lake Ljusvattentjärn (LJE) is surrounded by partially managed coniferous forest catchment, it covers an area of 0.02 km^2^ and has two basins which both have a maximum depth of 9 m. Erssjön (ERS) and Venasjön (VEN) are both situated in mixed agricultural and forest catchments and cover area of 0.06 km^2^ and 0.68 km^2^, respectively. Parsen (PRS) has 0.13 km^2^, and it is surrounded by coniferous forest.

### Sampling.

Sampling was conducted during the ice-free period for 133 days in total considering all lakes. In LJE, 43 days during the stratification period were covered (July 11–August 22, 2017). In ERS, three distinct campaigns included in total 11 days of the mixing period: September 12–15, 2017; October 31–November 3, 2017; and November 27–30, 2017. VEN was sampled for 41 days during the summer stratification period (June 15–July 3, 2018 and August 23–September 13, 2018) and for 12 days during the fall mixing period (September 14–25, 2018). Measurements in PRS (fall mixing period) covered 27 days (September 27–October 27, 2018).

### CH_4_ Flux Measurements.

Depending on the system, fluxes were measured either with manual chambers (40) or with automated flux chambers (AFCs) (41). Locations and depths of the chambers in each lake are shown in *SI Appendix*, Fig. S1. In ERS, 18 manual floating, horizontally anchored chambers (11, 42) were distributed over the lake at different depths (0.4 m to more than 1.5 m) to account for spatial variability. The type of chamber used has previously been shown to generate similar fluxes and gas transfer rates as approaches without enclosures, and the submerged depth of the chamber walls (∼3 cm) allows water turbulence to be transferred under the chambers while a low weight and horizontal mooring allows the chamber to follow wave motions (40, 42, 43). Gas from the manual chambers in ERS was collected at 2.9- to 8.9-h intervals by syringe and transferred to 22-mL tightly closed glass vials (precapped vials were flushed completely with 150 mL of the collected chamber gas samples and filled with 10 mL overpressure until just before analysis). Concentration of CH_4_ in vials was measured using gas chromatography following the procedure of Natchimuthu et al. (44). The CH_4_ fluxes were calculated based on the concentration changes in the chamber headspace as a function of time according to Bastviken et al. (6).

For the other lakes (VEN, PRS, LJE), 17 AFCs were used. Depending on the lake, the AFCs were distributed over different depths (0.9–7.8 m) due to the known influence of depth on CH_4_ emissions (6). Each AFC was equipped with a low-cost commercially available CH_4_ sensor (Figaro NGM 2611-E13; ref. 45), which measured in situ chamber headspace gas concentration every minute. Chambers were deployed over ∼0.4- to 3.1-h cycles; after every cycle of gas accumulation, the chamber automatically opened for 20 min to flush the chamber with ambient air. Additionally, to obtain data necessary for CH_4_ sensor calibration, in situ measurements of relative humidity (RH) and temperature in the chamber headspace of each AFC were recorded with K33 ELG sensors (SenseAir) (46). Prior to calculations, data were screened and checked, retaining only quality approved data (e.g., removing data affected by power failures or incomplete data logging). For this reason, measurements collected in July and part of August 2018 in VEN had to be removed as they did not meet the quality check. In case of missing temperature and RH for a given AFC (due to K33 ELG sensor malfunction), values of temperature and RH from the closest AFC and closest in time were used, assuming the same temperature and RH in both chambers. Afterward, each CH_4_ sensor was calibrated separately by following a procedure described in detail in Bastviken et al. (45) using background air concentrations under different humidity and well mixed atmospheric conditions (wind speed higher >2 m/s) as reference data. Finally, including volume and area of the chamber, CH_4_ flux (μmol·m^−2^·h^−1^) was calculated as a relative change in CH_4_ levels in a chamber over time.

### Environmental Drivers.

Water temperature in PRS, VEN, and ERS was measured at 1-Hz frequency with RBR thermistors (RBR*solo*^2^), which were placed well shaded at ∼0.1–0.2 m under the water surface. In LJE, the water temperature was collected every 5 min with TidbiT Water Temperature Data Loggers (Hobo, Onset). Meteorological variables (air temperature, air pressure, and wind speed at 10-m height) were obtained from the national meteorological analysis model (MESAN), whereas PAR was retrieved from a mesoscale model for solar radiation (STRÅNG), both available from the Swedish Meteorological and Hydrological Institute and driven by high-resolution real data from the national Swedish weather monitoring (smhi.se). Meteorological variables and PAR data were obtained as hourly averages.

### Data Analysis.

All of the analysis, including data processing, calculations, sensor calibrations, statistical tests, and figures, were performed with R studio (1.1.463) using packages broom, data.table, dplyr, ggplot2, ggpubr, plotly, psych, RColorBrewer, readxl, scales, vegan, and xlsx (47–58). To test for significant differences between respective data groups (day versus night fluxes), the nonparametric Wilcoxon test was applied.

Day:night FL_R_ for CH_4_ flux and wind speed in VEN, PRS, and LJE were calculated for each lake, each depth and each day-night cycle separately. In ERS, chambers were grouped according to depth range, resulting in four categories (<0.5 m, 0.5–1 m, 1–1.5 m, >1.5 m). All of the FL_R_ and wind speed values were calculated as the weighted average of the given variable measured during the day divided by the weighted average measured during the following night, where the length of each flux time period was used when calculating total flux. The length of the days and nights were defined according to sunrise and sunset times at each location and each day separately; each day-night cycle started with a sunrise and ended with a sunrise on a consecutive day, thus the length of each light vs. dark period was dependent on the lake and on the season.

To evaluate if pressure drops could potentially impact FL_R_, changes in atmospheric pressure were calculated. The highest pressure change was defined as the maximum negative slope derived from linear regression analysis fitted over moving 4-h time periods during each daytime and nighttime period. For the days when negative slopes were observed during nighttime and daytime, day:night pressure drop ratios were calculated.

Hourly averages of CH_4_ flux over the diel cycle as reported in Fig. 1 were calculated as the time-weighted geometric average of all fluxes measured during each hour of the day for each lake and season separately. The weights were defined as the length of each CH_4_ flux during each hour time interval. The geometric average was selected as the most optimal way to represent central tendency of emissions (59) in a diel cycle as it limits the influence of an extreme single values (i.e., occasional CH_4_ ebullition), which otherwise would elevate the whole average (60). The day and night averages for ΔT (*SI Appendix*, Fig. S2), wind speed (*SI Appendix*, Fig. S3), and PAR (*SI Appendix*, Fig. S5) were calculated the same way.

To derive the diel factor, the average daily flux, which in Wik et al. (35) was based on daytime measurements only, was used to back calculate the nighttime emission assuming 2.5 lower rate during the night. Further, new adjusted full diel fluxes were obtained as an average of the previous daytime flux and newly estimated nighttime emissions. This was calculated separately for each study referenced in Wik et al. (35), which did not account for diel variability. Finally, the ratio of 0.7 was obtained by dividing the updated flux by the flux reported in Wik et al. (35).

## Supplementary Material

Supplementary File

## Data Availability

Data associated with this study are available at Linköping University, liu.diva-portal.org/smash/record.jsf?pid=diva2%3A1440238&dswid=8377.
