# Supplementary material for: Diel variability of methane emissions from lakes
Source: Proc Natl Acad Sci U S A. 2020 Aug 17;117(35):21488–94. doi: 10.1073/pnas.2006024117 (PMC7474682; doi:10.1073/pnas.2006024117)
Supplement: Supplementary File [file pnas.2006024117.sapp.pdf]

PNAS
www.pnas.org

Supplementary Information for

Diel variability of methane emissions from lakes

Anna K. Sieczko, Nguyen T. Duc, Jonathan Schenk, Gustav Pajala, David Rudberg, Henrique O. Sawakuchi, David Bastviken

Anna Sieczko

Email: anna.sieczko@liu.se

This PDF file includes:

Figures S1 to S6

Tables S1 to S2

SI References

www.pnas.org/cgi/doi/10.1073/pnas.2006024117

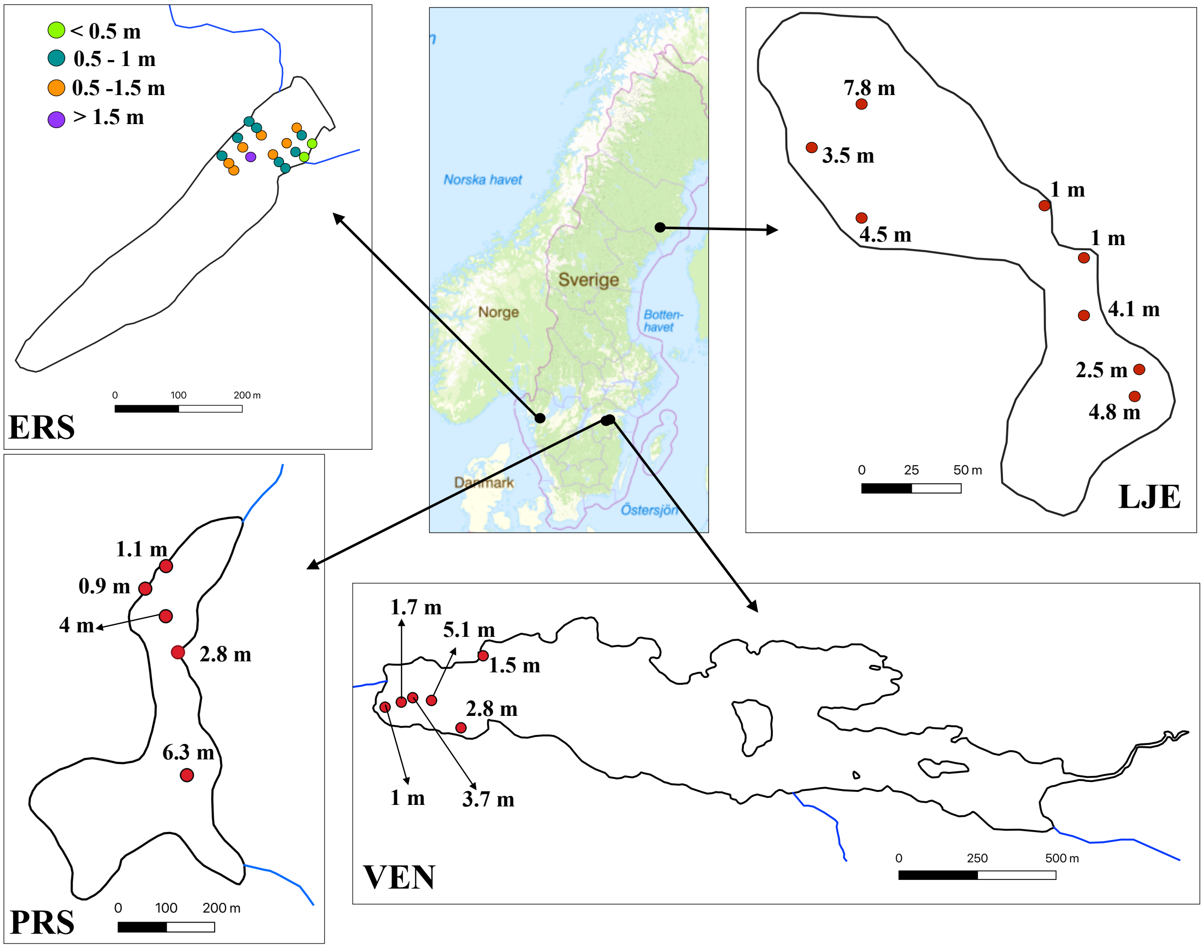

Fig. S1. Maps of the studied lakes: Ljusvatterntjärn (LJE), Erssjön (ERS), Parsen (PRS) and Venasjön (VEN). Red points indicate locations of the automated floating chambers. Nearby numbers indicate depth. Points at ERS indicate locations of the floating chambers which were grouped into depth categories. Colors indicate four different depth categories. Lantmäteriet. Geodataportalen, URL: https://www.geodata.se/geodataportalen, [retrieved between 2020-01-31 - 2020-02-25]

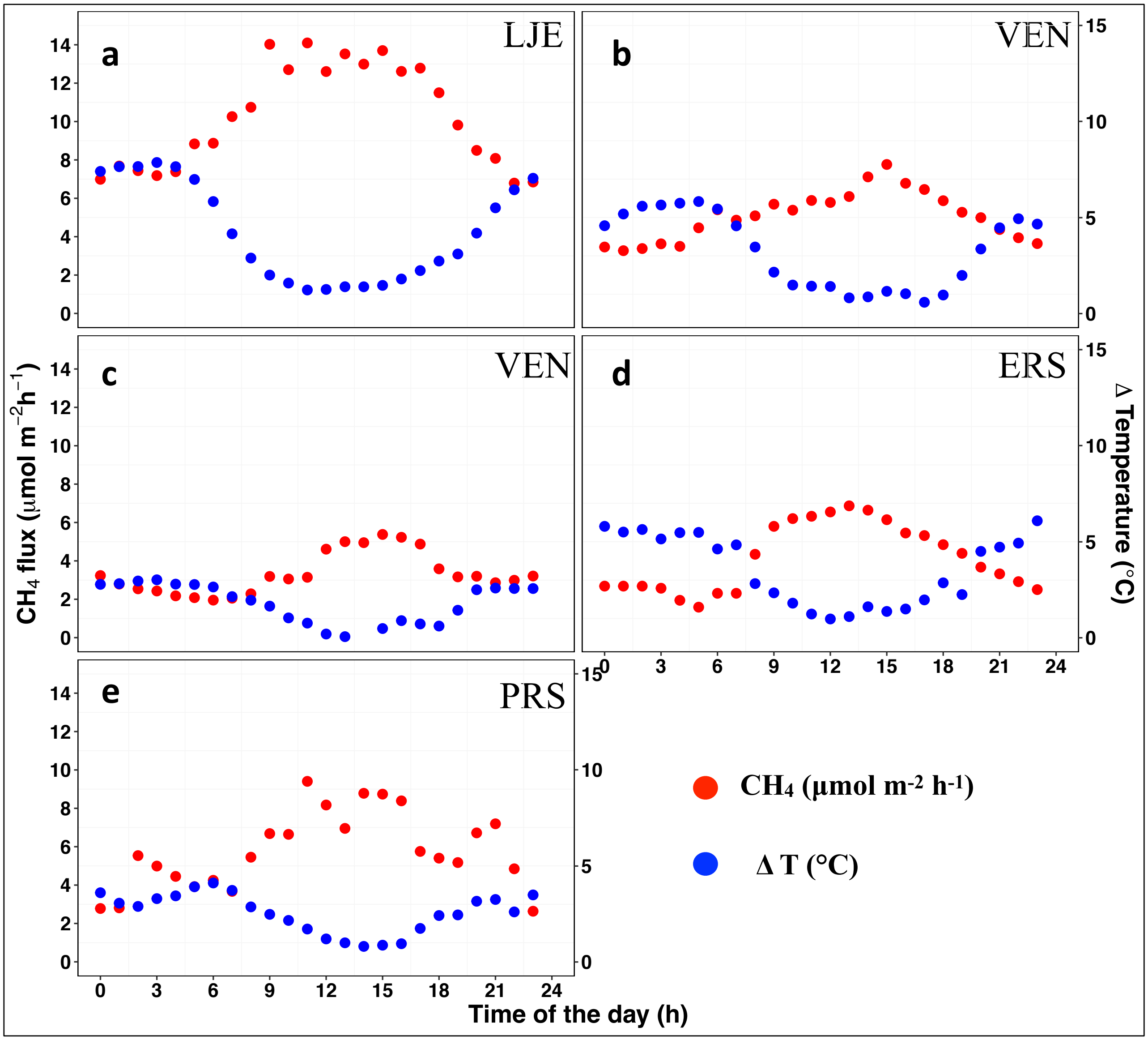

Fig. S2. Mean (geometric weighted average) of diel CH_4 flux patterns and the difference between water and air temperature in each lake during stratification (a, b) and mixing (c, d, e) periods. For abbreviations of lake names see Methods.

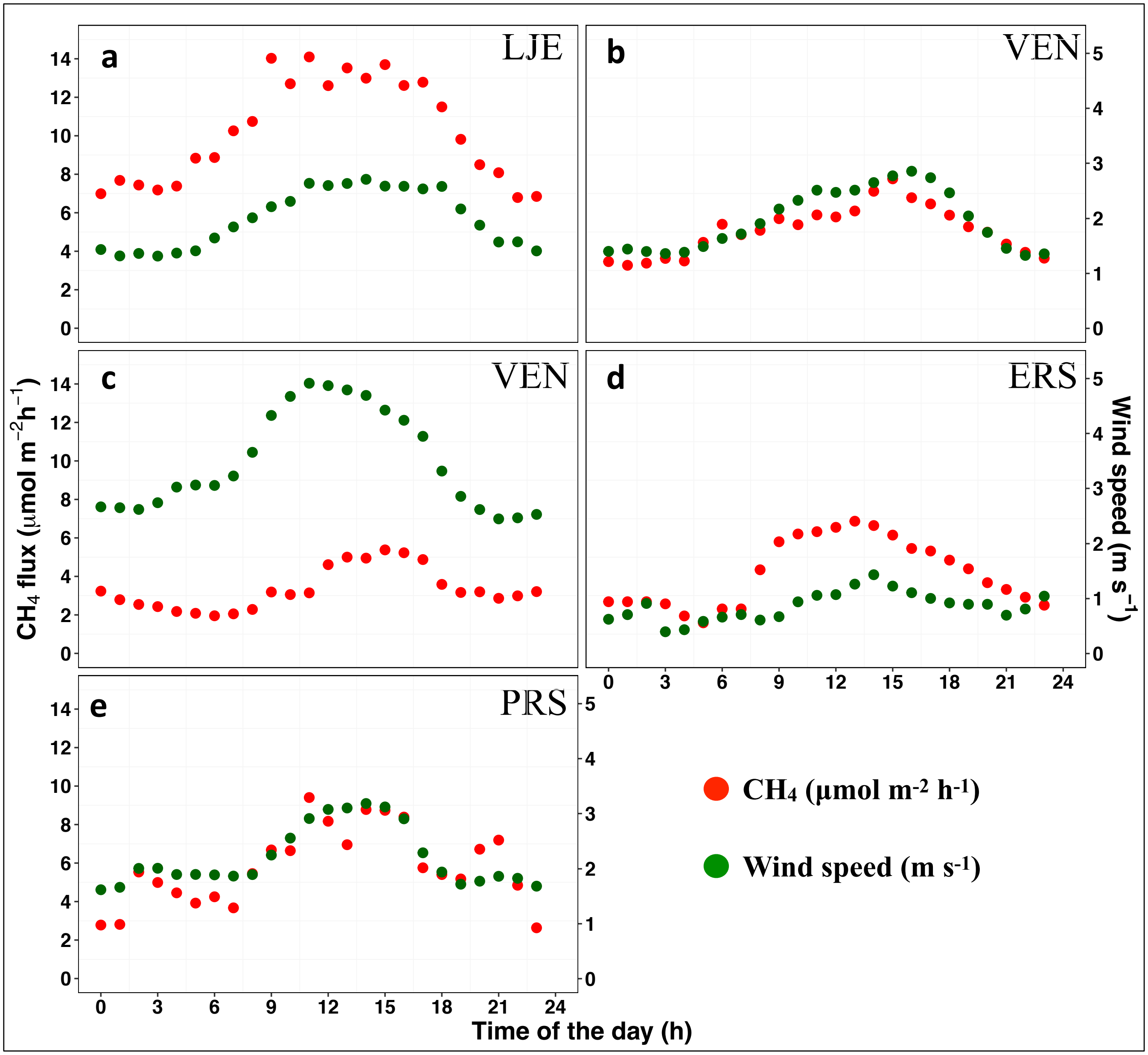

Fig. S3. Mean (geometric weighted average) of diel CH_4 flux patterns and wind speed in each lake during stratification (a, b) and mixing (c, d, e) periods. For abbreviations of lake names see Methods.

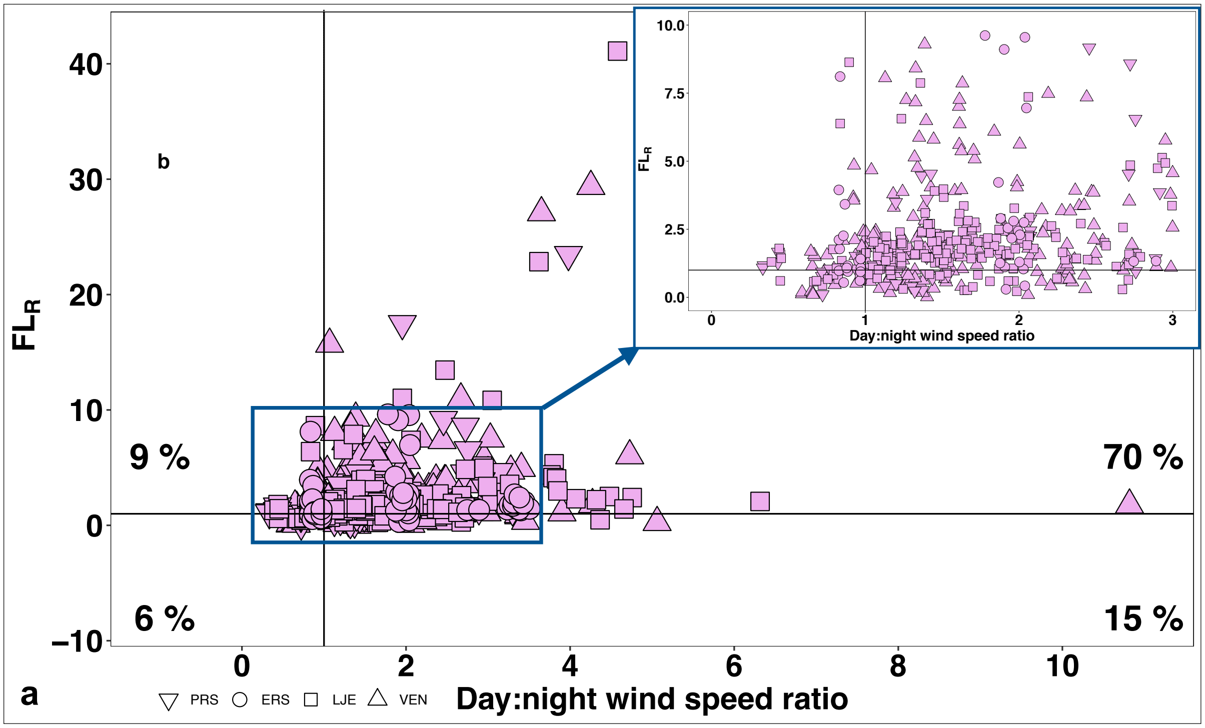

Fig. S4. CH_4 flux day:night ratio (FL_R) in the lakes investigated in this study plotted against day:night wind speed ratio for each chamber and day-night cycle in each lake (*n*=524). For abbreviations see Methods.

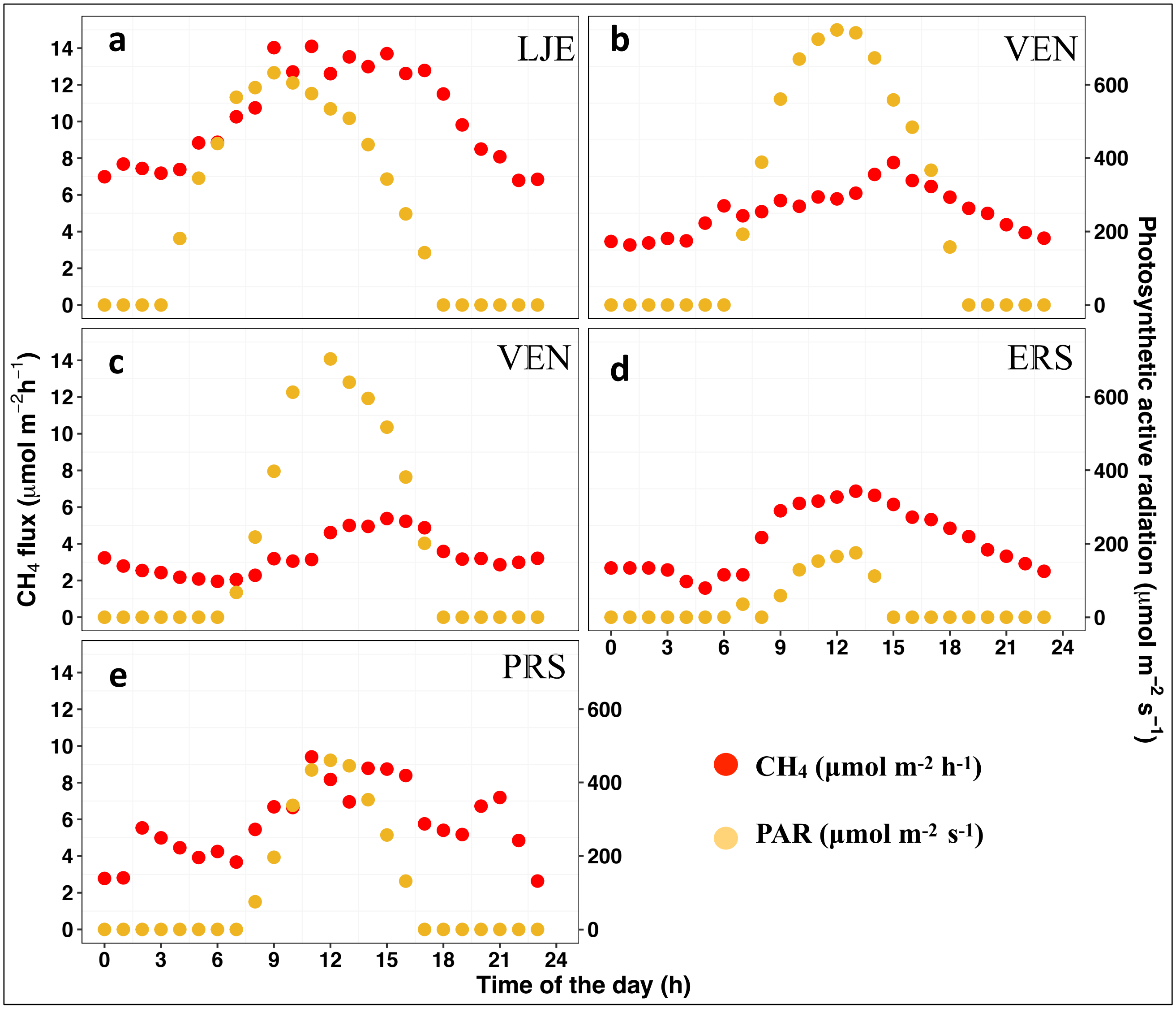

Fig. S5. Mean (geometric weighted average) of diel CH_4 flux pattern and photosynthetic active radiation in each lake during stratification (a, b) and mixing (c, d, e) periods. For abbreviation of lake names see Methods.

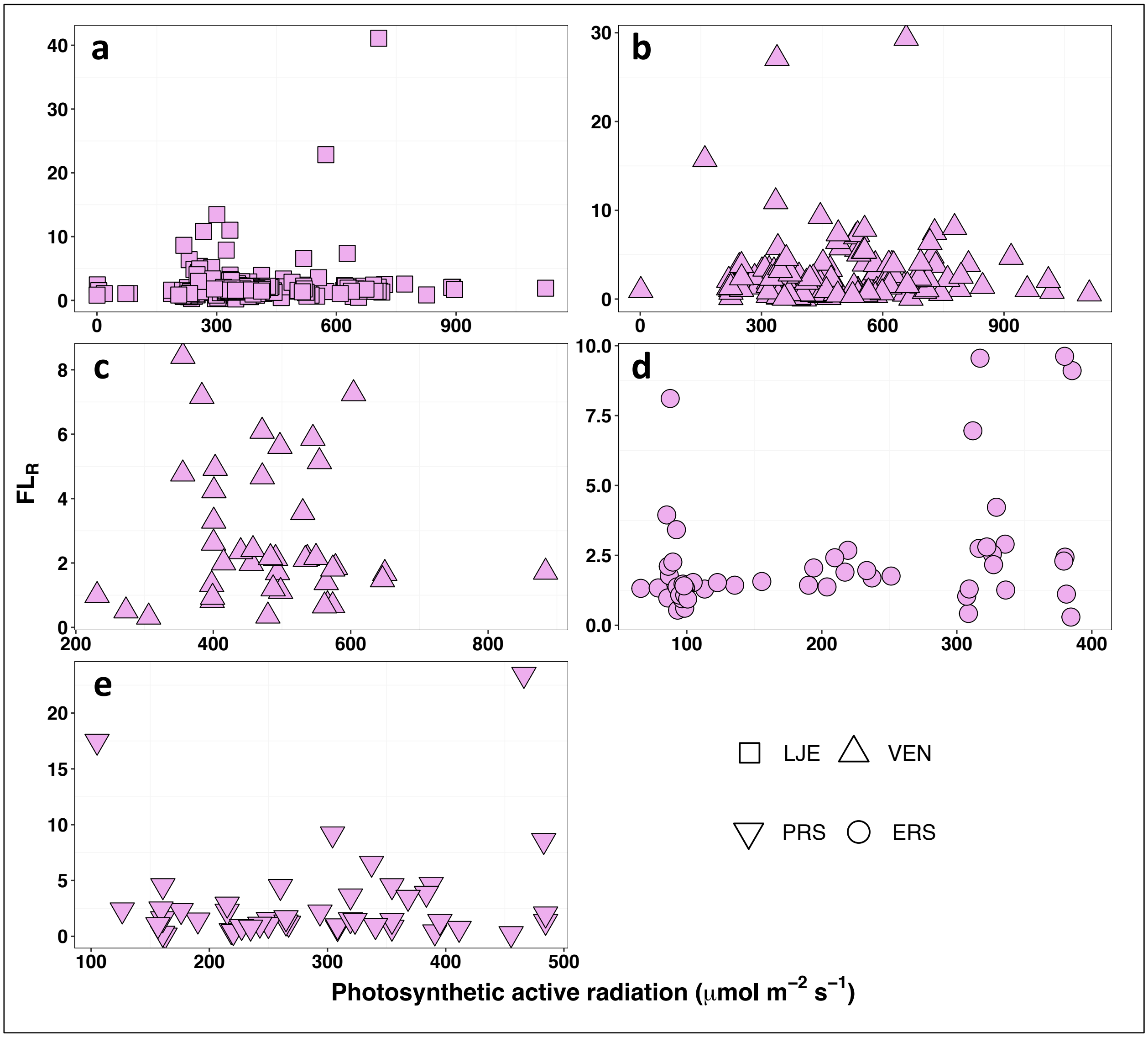

Fig. S6. CH_4 flux day:night ratio (FL_R) in the lakes investigated in this study plotted against average daytime photosynthetic active radiation (PAR) for each chamber and in each lake during stratified (a, b) and mixing (c, d, e) periods (*n*=524). For abbreviations of lake names see Methods.

Table S1. Main characteristics of the studied lakes with the mean and range (minimum-maximum) of selected parameters. For lake name abbreviations see Methods and Fig. S1. All the values are for the surface water (0.1 – 0.5 m). Total nitrogen (TN); total phosphorus (TP); dissolved organic carbon (DOC); chlorophyll a (chl *a*).

Parameter	LJE	VEN	ERS	PRS
Coordinates	64°05'34.0"N 18°55'43.0"E	58°27'22.0"N 16°10'31.0"E	58°22'23" N, 12°89'55" E	58°20'39.0"N 16°12'23.0"E
Water temperature (°C)	15.0 – 20.0 (18.0)	12.9- 20.7 (16.8)	2.0 – 17.7 (7.6)	6.6 – 12.4 (10.3)
TN (mg l^{-1})	0.15 – 0.42 (0.25)	0.41 – 0.96 (0.78)	0.7 – 1. 4 (1.1)*	0.59 – 0.6 (0.62)
TP (µg l^{-1})	6.9 – 12.9 (9.9)†	38.1 – 64.7 (48.7)	20.6 – 60.1 (35.0)*	16.6 – 17.5 (17.1)
DOC (mg l^{-1})	6.3 - 22.9 (12.2)	13.4 – 18.5 (14.9)	15.8 – 27.7 (22.5)*	14.6 – 17.7 (15.7)
pH	5.7-6.5 (6.2)†	7.3-8.1 (7.6)	3.9 - 6.8 (5.0)	6.4-7.1 (7.0)
Conductivity (µS cm^{-1})	8-16 (12)†	161-188 (170)	65 – 261 (103)	72-92 (78)
Max depth (m)	9.4	11	4.5	6.7
Chl *a* (µg l^{-1})	1.1-1.6 (1.3)	17.8-42.1 (29.8)	0.2-16.9 (4.6)	2.5-4.3 (3.4)

* Data from Natchimuthu et al. 2016 (1); †average for May-July 2019.

Table S2. Examples of studies on diel variability of CH_4 fluxes in lakes, ponds and reservoirs

	**study**	**Ecosystem type**	**Method**	**Season**	**Frequency of flux**	**Duration of the study**	**Highest flux**	**Mean day/night ratio**
FLUX CHAMBER	Bastviken et al. (2004) (2)	Temperate **lakes**	Floating chambers	summer	1 – 8 times a day	1 day	day	1.7
	Bastviken et al. (2010) (3)	Tropical floodplain **lake**	Floating chambers	low water	4 times a day (every 10th minutes for 40 minutes)	1 day	Afternoon (15:00 – 16:00)	2
	Liu et al. (2017) (4)	Subtropical lake	Floating chambers	wet	2 h	1 day	late morning (12:00)	-11.3
				wet	2 h	1 day	late morning (12:00)	8.7
				dry	2 h	1 day	Pre-dawn	4.6
				dry	2 h	1 day	late morning (12:00)	7.6
			Floating chambers	autumn stratified	2 – 3 times a day	11 days	day	2.2
	Erkkila et al. (2018) (5)	Boreal **lake**	Floating chambers	autumn stratified	2 – 3 times a day	11 days	day	2.2
	Sieczko et al. (2020) (*this study)*	Boreal **lakes**	Automated flux chambers	summer	0.5 – 3.1 h	43 days	day	2.3
					0.4 – 2.9 h	40 days		2.7
				autumn	0.4 – 2.8 h	12 days		2.7
					0.4 – 2.7 h	27 days		2.9
			Floating chambers	autumn	2.9 – 8.9 h	11 days		2.4
	Xiao et al. (2013) (6)	Subtropical hydropower **reservoir**	Dynamic closed chamber (cavity ringdown spectroscopy)	wet and dry	0.5 h	3 days in total (1 day each month)	no clear pattern	not reported

	Linkhorst (2019) (7)	Tropical **reservoir**	Floating chambers + bubble traps		Every 1 h for 5 minutes (x3)	1 day (48 h)	day	~1.01
		Tropical **reservoir**			Every 1 h for 5 minutes (x3)	1 day (48 h)	day	~2.3
	Xiao et al. (2014) (8)	Subtropical **pond**	Static closed chamber (cavity ringdown spectroscopy)	summer, autumn	0.5 h	3 days (2+1)	night	0.81
	Bansal et al. (2018) (9)	Wetland **pond**	automatic chamber	spring, summer, autumn	6 – 9 times a day	11 months over 2 years	day	not reported
EDDY COVARIANCE	Podgrajsek et al. (2014) (10)	Boreal **lake**	Eddy covariance	spring	0.5 h	4 days	night	~ 0.2
				autumn	0.5 h	10 days	night	~ 0.3
	Franz et al. (2016) (11)	Temperate peatland **lake**	Eddy covariance	spring, summer, autumn, winter	0.5 h	1 year	night	not reported
	Xiao et al. (2017) (12)	subtropical **lake**	flux gradient and the eddy covariance method	spring, summer, autumn, winter	0.5 h	1.9 year	night	0.95
	Jammet et al. (2017) (13)	Subarctic peatland **lake**	eddy covariance	summer	24	3 x 4.5 month	morning	not reported
	Erkkila et al. (2018) (5)	Boreal **lake**	eddy covariance	autumn stratified	0.5 h	11 days	day	1.2
				autumn mixing	0.5 h	5 days	day	1.2

	Sollberger et al. (2017) (14)	Alpine hydropower **reservoir**	eddy covariance	spring, summer, autumn	0.5 h	8.7 month	day	3.2
**GAS TRANSFER MODEL**	Ford et al. (2002) (15)	Floodplain **lake**	surface water concentration	autumn	4 times a day	3.5 days	morning	18
			surface water concentration	summer	6-10 times a day	3.5 days	morning	2
	Erkkila et al. (2018) (5)	Boreal **lake**	*k* model (Cole and Caraco 1998)	autumn stratified	2 h	11 days	night	0.7
				autumn mixing	2 h	5 days	day	1.3
			k model (Tedford et al. 2014)	autumn stratified	2 h	11 days	night	0.8
				autumn mixing	2 h	5 days	day	1.1
			k model (Heiskanen et al. 2014)	autumn stratified	2 h	11 days	night	0.4
				autumn mixing	2 h	5 days	day	1.1
			surface water concentration	summer	6-10 times a day	3.5 days	morning	2

SI References

1. S. Natchimuthu *et al.*, Spatio-temporal variability of lake CH_4 fluxes and its influence on annual whole lake emission estimates. *Limnol. Oceanog.* **61**, S13-S26 (2016).
2. D. Bastviken, J. Cole, M. Pace, L. Tranvik, Methane emissions from lakes: Dependence of lake characteristics, two regional assessments, and a global estimate. *Global Biogeochem. Cycles* **18** (2004).
3. D. Bastviken *et al.*, Methane emissions from Pantanal, South America, during the low water season: toward more comprehensive sampling. *Environ. Sci. Technol.* **44**, 5450-5455 (2010).
4. L. Liu, M. Xu, R. Li, R. Shao, Timescale dependence of environmental controls on methane efflux from Poyang Hu, China. *Biogeosciences* **14**, 2019-2032 (2017).
5. K.-M. Erkkila *et al.*, Methane and carbon dioxide fluxes over a lake: comparison between eddy covariance, floating chambers and boundary layer method. *Biogeosciences* **15**, 429-445 (2018).
6. S. Xiao, D. Liu, Y. Wang, Z. Yang, W. Chen, Temporal variation of methane flux from Xiangxi Bay of the Three Gorges Reservoir. *Sci.Rep.* **3**, 2500 (2013).
7. A. Linkhorst, Greenhouse gas emission from tropical reservoirs: Spatial and temporal dynamics. (2019).
8. S. Xiao *et al.*, Gas transfer velocities of methane and carbon dioxide in a subtropical shallow pond. *Tellus B* **66**, 23795 (2014).
9. S. Bansal, B. Tangen, R. Finocchiaro, Diurnal patterns of methane flux from a seasonal wetland: Mechanisms and methodology. *Wetlands* **38**, 933-943 (2018).
10. E. Podgrajsek, E. Sahlée, A. Rutgersson, Diurnal cycle of lake methane flux. *J. Geophys. Res. Biogeosci.* **119**, 236-248 (2014).
11. D. Franz, F. Koebsch, E. Larmanou, J. Augustin, T. Sachs, High net CO_2 and CH_4 release at a eutrophic shallow lake on a formerly drained fen. *Biogeosciences* **13**, 3051-3070 (2016).
12. Q. Xiao *et al.*, Spatial variations of methane emission in a large shallow eutrophic lake in subtropical climate. *J. Geophys. Res. Biogeosci.* **122**, 1597-1614 (2017).
13. M. Jammet *et al.*, Year-round CH_4 and CO_2 flux dynamics in two contrasting freshwater ecosystems of the subarctic. *Biogeosciences* **14**, 5189-5216 (2017).
14. S. Sollberger, B. Wehrli, C. J. Schubert, T. DelSontro, W. Eugster, Minor methane emissions from an Alpine hydropower reservoir based on monitoring of diel and seasonal variability. *Environ. Sci. Process. Impacts* **19**, 1278-1291 (2017).
15. P. W. Ford, P. I. Boon, K. Lee, Methane and oxygen dynamics in a shallow floodplain lake: the significance of periodic stratification. *Hydrobiologia* **485**, 97-110 (2002).
